# Toward a Theory of Proactive Health-Oriented Livestock Production: A Testable Conceptual Framework for Alpine Pastoral Systems

**DOI:** 10.3390/life16091509

**Published:** 2026-09-10

**Authors:** Wenhao Li, Yuan Li, Qi-Tala An

**Affiliations:** 1 College of Animal Science and Veterinary Science, Qinghai University, Xining 810016, China; qhdxlwh@163.com (W.L.); quan10242024@icloud.com (Y.L.); 2 Qilian Tibetan Sheep Industrial Development Research Institute of Qinghai Province, Qilian 810400, China

**Keywords:** proactive health-oriented livestock production, conceptual framework, risk-triggered intervention, alpine pastoral systems, Tibetan sheep, yak, One Health, animal welfare

## Abstract

Livestock production must reconcile animal health, food safety, ecological limits, and farm viability. This narrative review proposes proactive health-oriented livestock production (PHLP) as a testable conceptual framework for alpine pastoral systems and as a step toward theory development, rather than as an already validated general theory. PHLP does not claim novelty for vaccination, nutrition, welfare, biosecurity, precision monitoring, or low-carbon practices considered separately. Its proposed contribution is a decision architecture that forecasts context- and stage-specific risk, detects deviation with a feasible sentinel dataset, activates a matched intervention before avoidable loss becomes clinically or economically visible, and recalibrates local triggers from animal, product, ecosystem, and livelihood outcomes. The hypothesized pathway links cold, hypoxia, forage scarcity, pathogen exposure, and management stress to sentinel change, trigger-based action, lower cumulative stress, and greater immune–metabolic, rumen–gut, and behavioral stability. We define entry points, priority rules, initial boundary conditions, measurable indicators, and three testable propositions for Tibetan sheep, yak, and other alpine ruminants. PHLP is expected to be most useful when a consequential risk has usable lead time, a sentinel variable can be observed, and a feasible action is available; it complements rather than replaces emergency clinical care and statutory disease control. Comparative longitudinal and multisite studies are needed to calibrate triggers and test whether PHLP improves decision timeliness and reduces seasonal body-condition loss, morbidity, mortality, antimicrobial treatment, product-quality variability, and emission intensity relative to reactive or calendar-based management.

## 1. Introduction

Livestock production supplies nutrient-dense animal-source foods, supports pastoral livelihoods and shapes the stability of grassland ecosystems. Consumer demand is shifting from volume alone toward food safety, nutritional quality and sustainable supply. At the same time, animal disease, antimicrobial stewardship, farm pollution, greenhouse gas emissions and animal welfare are increasingly interconnected. Health-oriented livestock production has therefore become a systems challenge spanning animals, products, ecosystems and farm viability.

Proactive health moves management upstream by identifying and modifying risks before disease or functional impairment becomes evident, and by maintaining this approach throughout life [1,2]. In the present article, proactive health is treated as a timing and decision principle rather than as a novel veterinary intervention. This logic aligns with One Health [3], incorporates welfare as both a sentinel and safeguard [4,5], and can use precision livestock farming tools [6,7,8], biosecurity [9], antimicrobial stewardship [10,11], and low-carbon practices [12,13]. Applied to livestock, PHLP does not displace vaccination, statutory disease control, or clinical care. It asks when risk becomes actionable, which animals or stages should be prioritized, which feasible action fits the dominant risk, and how outcomes should recalibrate later decisions.

The Qinghai–Tibet Plateau supports ecologically important grasslands and is the principal production region for Tibetan sheep, yak and other indigenous ruminants. Long exposure to cold, hypoxia, intense ultraviolet radiation and seasonal forage variability has shaped genetic, metabolic, phenotypic, and microbial characteristics in these animals [14,15,16,17,18]. These studies define the biological context in which management operates; they do not by themselves demonstrate that a particular management intervention causally improves adaptation or health. Biological adaptation is therefore distinguished here from management adaptation: the former comprises inherited or acquired animal traits, whereas the latter comprises decisions that alter exposure, nutrition, surveillance, or care. Adaptation also does not eliminate winter and spring forage deficits, body condition loss, environmental stress, subclinical infection, or inappropriate veterinary drug use. Mismatches between stocking pressure and forage supply and inconsistency in product quality continue to constrain many alpine pastoral systems.

Most health management technologies were developed in intensive farms or low-altitude agricultural regions and therefore require recalibration before use in alpine grazing systems. Thermal comfort, ventilation, and housing set points derived from confined animals do not capture wind chill, large diurnal temperature ranges, solar radiation, or animal movement across heterogeneous terrain. Fixed rations and measured dry matter intake are difficult to maintain when animals select seasonally variable pasture. Sensor systems may assume continuous power, connectivity, individual identification, and stable behavioral baselines that dispersed grazing herds cannot provide. Manure recovery and emission control technologies designed for housed systems likewise assume collectable waste streams. These are not simply different environments; they are different measurement, control, and implementation conditions.

These constraints call for a framework that combines proactive timing with plateau ecology and the physiology of indigenous livestock. This article therefore proposes PHLP as a testable conceptual framework, traces its origins, defines its decision rules and boundary conditions, and derives operational pathways and research propositions. The aim is not to rename established good practice, but to specify how context, life stage, sentinel signals, action triggers, matched interventions, and multi-level outcomes can be linked in alpine ruminant production.

## 2. Review Scope and Conceptual Development

This article is a narrative, theory-building review rather than a systematic review or meta-analysis. The literature was used to identify established concepts, characterize the constraints of alpine pastoral production, and examine component-level evidence relevant to Tibetan sheep, yak, and comparable ruminant systems. Regional and species-specific studies were prioritized when available. Evidence from other production systems is used only as proof-of-principle and is identified as such; it is not treated as direct validation of plateau-specific thresholds or of the integrated PHLP framework.

Framework development followed four steps. First, proactive health, preventive veterinary medicine, One Health, animal welfare, precision livestock farming, and sustainable livestock production were separated by their unit of decision, trigger, and objective. Second, biological and managerial constraints of alpine grazing systems were mapped across breeding, growth, reproduction, grazing, transport, product formation, and nutrient return. Third, recurrent decision failures were translated into a common sequence of risk forecast, sentinel observation, locally calibrated trigger, matched action, outcome assessment, and feedback. Fourth, initial boundary conditions, candidate indicators, and directional propositions were specified so that the framework can be compared with reactive, calendar-based, or single-domain management. This procedure organizes a conceptual synthesis; it does not constitute empirical validation.

## 3. Conceptual Origins of Proactive Health and Its Translation to Livestock Science

Proactive health has emerged as a governance approach that shifts the point of intervention upstream. It seeks to preserve physiological stability through risk identification, behavioral intervention, environmental optimization and continuous monitoring before disease or functional loss occurs. Compared with clinically centered models, proactive health tracks changing risks, functional status and adaptive capacity across the life course [1,2]. Management consequently extends from treatment after onset to prevention, early detection and dynamic regulation.

From a public health perspective, proactive health intersects with health promotion, chronic disease prevention and life course management. One Health broadens this logic by recognizing the interdependence of human, animal and ecosystem health [3]. Livestock health should therefore be evaluated within its connections to food safety, public health, ecosystem integrity and socioeconomic development. It should not be defined simply by the absence of clinical disease. Growth, immune and metabolic status, behavior, environmental adaptability and production risk are also relevant dimensions.

Animal welfare science provides a necessary input to PHLP, but PHLP gives welfare a specific operational role. Behavioral deviation, altered resting or feeding patterns, social disruption, and exposure to avoidable discomfort are treated as sentinel signals of declining adaptive capacity, not only as ethical outcomes assessed after the event [4,5]. Welfare also acts as a decision constraint: a productivity gain is not considered an improvement if it depends on sustained hunger, thermal discomfort, restricted behavior, or excessive handling. Welfare indicators therefore function within PHLP as early-warning variables, plausible mediators between exposure and health, and safeguards against one-dimensional optimization; these roles do not imply that the underlying welfare concepts are new.

Precision livestock farming can make proactive management more operational by enabling repeated observation and earlier intervention [6,7,8]. PHLP, however, is technology-neutral. In resource-limited grazing systems, a minimum dataset can comprise body-condition trajectory, reproductive stage, local weather and forage forecasts, disease history, treatment records, and structured observation of intake, movement, and behavior. Enhanced systems may add group weighing, GPS trajectories, or portable temperature measurements, whereas advanced systems may use individual sensors and automated alerts. Tool selection should therefore follow the decision need, infrastructure, maintenance capacity, false alert burden, and farmer usability rather than assuming that the most complex sensor is the most appropriate.

Introducing proactive health into livestock production does not replace established prevention or husbandry. Instead, it reorganizes when and how these established measures are deployed. The underlying premise is that livestock health is dynamic and emerges from interactions among genetics, nutrition, environment, microbiota, immunity, behavior and management. Its proposed contribution lies in the organization and testability of decisions across domains, not in claiming that the component practices themselves are new.

## 4. Evolution of Health-Oriented Production and Constraints in Alpine Pastoral Systems

In China, health-oriented livestock production developed alongside industrial scaling, farm standardization and food-safety regulation. Early programs focused on infectious disease control, feed safety, responsible veterinary drug use and environmental improvement [19,20,21]. Their immediate targets were high disease incidence, low productivity and variable product quality. As food safety and sustainability expectations increased, the concept expanded beyond the absence of disease. It now also encompasses process control, product safety, environmental performance and economic viability.

Compared with low-altitude agricultural regions, alpine pastoral systems are more tightly constrained by climate and grassland resources [22,23]. Cold, hypoxia and intense ultraviolet radiation impose chronic physiological demands. Although Tibetan sheep and yak are well adapted, winter forage deficits and wide diurnal temperatures can still reduce body condition, reproductive performance and juvenile survival. Grazing itself also carries an appreciable energetic cost, particularly during cold or severe weather. Seasonal forage growth creates further mismatches between nutrient supply and physiological demand. Dispersed herds complicate vaccination, surveillance, clinical care and standardized management, weakening early detection of disease and subclinical infection. Excessive stocking, poor rangeland management and unmanaged manure can further intensify land degradation and disrupt nutrient cycling.

These conditions expose three important limitations in generic livestock health management. First, action is often delayed until clinical signs, weight loss or production decline become visible, which is poorly suited to predictable cold season undernutrition, transport stress, grazing stress, or subclinical dysfunction. Second, intensive system assumptions may not hold: individual feed intake is rarely measured on pasture; weather and forage exposures vary within a grazing day; herds are dispersed; power and connectivity are intermittent; and confinement-derived behavioral thresholds may not identify risk in free-ranging animals. Third, measures are often designed in isolation, with limited coordination among animal health, rangeland condition, emissions, product quality, and pastoral income.

Alpine pastoral regions benefit from natural grazing systems, indigenous breeds, and strong potential for certified green or organic production. These assets, however, do not automatically yield consistent quality or market value. A management system must be locally adapted, measurable, and economically feasible to convert ecological endowments into durable product and livelihood benefits. PHLP addresses this need by placing anticipatory intervention, continuous feedback, and multiple production goals within one explicitly bounded decision architecture.

## 5. Definition, Distinctive Contribution, and Operating Logic of PHLP

### 5.1. Theoretical Position and Distinctive Contribution

Proactive health, One Health, animal welfare and precision livestock farming each contribute a distinct perspective on livestock health. Our previous work explored proactive health practices and environmentally sound forage production systems in alpine pastoral areas of Qinghai [24,25]. These regional experiences informed the conceptual synthesis developed in the present article. PHLP is proposed here as a testable conceptual framework and as a step toward theory development, rather than as a discrete technology or a repackaged set of interventions. It organizes health objectives and interventions throughout the life cycle of alpine livestock.

We define PHLP as a whole-life-cycle, risk-triggered framework that coordinates established veterinary, husbandry, welfare, and ecological measures before avoidable loss becomes clinically or economically visible. It evaluates success across animal health, product quality and safety, resource use, ecosystem condition, and farm viability. Preventive veterinary medicine remains an essential module within PHLP; PHLP extends the unit of decision from specific disease prevention to interacting infectious, nutritional, environmental, behavioral, production, and ecosystem risks, and extends feedback from disease occurrence to linked animal product–ecosystem–livelihood outcomes.

Component-level evidence relevant to this architecture includes cold season feeding strategies in Tibetan sheep and yak [26,27], rotational and deferred-grazing or restoration regimes on the Qinghai–Tibet Plateau [28,29], body condition management between pregnancy scanning and lambing in commercial sheep systems [30], activity and rumination as preclinical signals in cattle [31], multi-trait sheep breeding objectives [32], and low-temperature sheep transport in northern China [33]. These studies justify individual links or implementation examples; none validates the entire PHLP sequence as a single causal system. The positioning of PHLP relative to adjacent frameworks is summarized in Table 1.

**Table 1:** Positioning of PHLP relative to adjacent frameworks.

Framework	Primary Unit or Trigger	Primary Objective	Role Relative to PHLP
Preventive veterinary medicine	Disease, pathogen, herd; epidemiological or scheduled trigger	Prevent infection and disease	Essential disease control module; PHLP also ranks nutritional, environmental, behavioral, product, and ecosystem risks.
One Health	Human–animal–environment interface	Coordinate cross-sector health consequences	Defines cross-system consequences; PHLP supplies an on-farm, life-stage decision sequence.
Animal welfare	Animal experience, behavior, and physical or affective state	Avoid harm and enable acceptable living conditions	Supplies sentinel indicators, plausible mediators, and non-negotiable safeguards.
Sustainable livestock production	Resource use and environmental, social, and economic performance	Maintain production within ecological and societal limits	Supplies outcome constraints; PHLP specifies when and how health-oriented action is triggered.
Precision livestock farming	Sensor-derived individual or group data	Monitor, predict, and automate management	Optional toolset; PHLP specifies the decision need and permits low-cost alternatives.
PHLP	Context-stage risk state; locally calibrated trigger	Act before avoidable loss and coordinate multi-level outcomes	Orders established measures through forecast, detection, matched action, assessment, and feedback.

### 5.2. Operating Mechanism, Entry Points, and Priority Rules

Four connected tenets organize this definition and distinguish PHLP from conventional health management approaches.

First, PHLP moves the point of management intervention upstream, before losses become apparent. Vaccination, disinfection, clinical treatment and culling remain essential for preventing infection and limiting disease losses. PHLP adds systematic attention to preclinical risk through breed–environment matching, forage planning, stress mitigation, behavioral alerts and pathogen surveillance. The objective is to reduce the probability that animals progress toward subclinical dysfunction or overt disease.

Second, PHLP uses anticipatory intervention to support physiological homeostasis under changing environmental conditions. Alpine livestock face sustained cold, hypoxia, nutritional fluctuation and pathogen pressure. Their health depends on the relative stability of immune, metabolic, microbial and behavioral systems. Proactive regulation does not mean maximizing material inputs or short-term productivity. It means matching nutrition, supplementation, rumen–gut management and environmental measures to physiological stage and anticipated exposure. This approach is intended to preserve resilience to environmental stressors.

Third, PHLP extends health management across the production chain and animal life cycle. Health outcomes accumulate through interacting genetic, nutritional, environmental, pathogenic and managerial influences. They cannot always be attributed to one pathogen or a single management failure. PHLP therefore links breeding, rearing, grazing, supplementation, vaccination, surveillance, transport, slaughter and manure management in an auditable chain with feedback between stages.

Fourth, PHLP balances production, environmental and livelihood outcomes within a common decision framework. Alpine livestock systems must remain economically viable while protecting grasslands, improving product quality, reducing veterinary drug use and limiting emissions. PHLP therefore treats animal health, ecosystem stability, farm performance and pastoral livelihoods as interdependent decision objectives.

**Figure 1:**
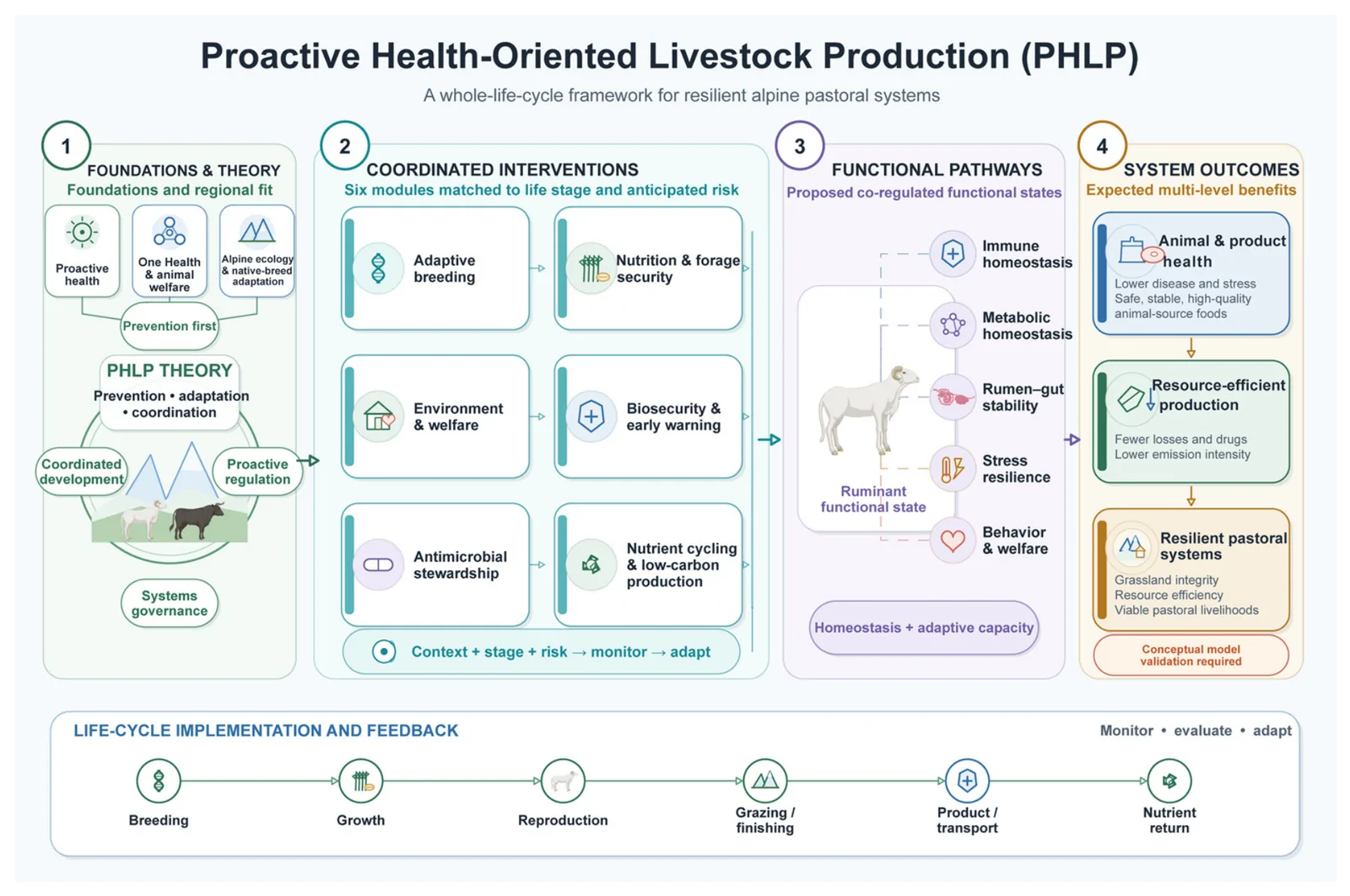
Conceptual framework for proactive health-oriented livestock production (PHLP) in alpine pastoral systems. The diagram links foundations and context to six whole-life-cycle intervention modules, proposed functional pathways, expected system outcomes, and life-cycle monitoring and feedback. In operational use, context and physiological stage define risk; feasible sentinel observations inform a locally calibrated action trigger; a matched intervention is then evaluated against intermediate and final outcomes. Arrows indicate proposed coordination and directional hypotheses rather than experimentally established causality. The same graphical framework is retained; numerical thresholds and effect sizes remain to be established through prospective local calibration.

Figure 1 presents the architecture of the hypothesized pathway. Foundations and regional constraints inform six whole-life-cycle intervention modules; these modules are proposed to influence immune–metabolic regulation, rumen–gut stability, stress resilience, and behavior and welfare; those intermediate states are expected to influence animal, product, ecological, and livelihood outcomes; and life-cycle monitoring provides feedback. Operationally, the pathway is read as risk forecast, sentinel observation, locally calibrated trigger, matched intervention, intermediate response, outcome assessment, and recalibration. The figure is a hypothesis map rather than evidence of established causality.

### 5.3. Boundary Conditions, Candidate Indicators, and Testable Propositions

PHLP is intended for alpine grazing or semi-grazing ruminant systems in which a consequential risk can be anticipated with usable lead time, at least one sentinel variable can be observed, and a feasible action can be taken before avoidable loss becomes evident. Local calibration is required because the same body condition trend, weather exposure, behavior change, or disease signal may have different predictive value among species, physiological stages, elevations, seasons, and production systems. The framework is not a substitute for emergency clinical care, outbreak control, or statutory veterinary action. Its incremental value is expected to be limited when risk is unpredictable, no feasible action exists, or intensive control already removes the relevant contextual uncertainty.

Candidate measures are deliberately presented as domains rather than universal cutoffs. Each validation study should specify the unit of observation, sampling frequency, local baseline, trigger derivation, comparator, expected direction, and decision consequence. Table 2 links PHLP components to measurable indicators. Numerical thresholds should be derived prospectively and recalibrated when sensitivity, specificity, cost, welfare, or implementation burden is unacceptable.

## 6. Technical Framework and Operational Pathways for PHLP

### 6.1. Adaptive Breeding as a Biological Foundation

Locally adapted genetic resources provide the biological foundation for PHLP. Tibetan sheep and yak have acquired tolerance to cold, hypoxia, low-quality forage and extensive grazing through natural and human selection. Genomic studies link high-altitude adaptation in Tibetan sheep to variants involved in hypoxia responses, erythrocyte function, energy metabolism and hemoglobin oxygen affinity [14,15]. Microbiome studies describe breed- and season-associated rumen characteristics relevant to energy extraction from low-quality forage [16,17]. A separate review summarizes feeding, growth, production, biochemical, and reproductive characteristics of yak under harsh plateau conditions [18]. These sources support biological plausibility and local calibration; they do not establish that a health management intervention causally improves biological adaptation. Management adaptation instead refers to changing breeding objectives, feed allocation, exposure, surveillance, or care in response to measured risk.

Breeding objectives should extend beyond body weight, meat yield, or reproductive rate to include stress resilience, disease resistance, feed use efficiency, cold season maintenance, maternal behavior, grazing ability, and product quality. These objectives cannot be maximized independently. Genetic correlations and economic weights can shift selection responses among growth, reproduction, carcass, wool, and parasite resistance traits [32]. PHLP therefore favors a locally weighted multi-trait index, conservation of within-breed diversity, and routine review of correlated responses rather than narrow selection for short-term output.

### 6.2. Nutritional Regulation and Forage Systems to Limit Seasonal Stress

Nutrition directly influences immune function, metabolism, reproduction and gut health. Forage quantity and quality vary sharply across seasons in alpine pastoral systems. Warm season herbage is relatively nutritious, whereas cold season forage contains more fiber and supplies less protein and energy. Grazing Tibetan sheep and yak can lose substantial live weight during the cold season, whereas oat hay, oat silage, total mixed rations, concentrate, or urea–molasses blocks can reduce loss or improve growth and rumen function under plateau conditions [26,27]. Late gestation, lactation, juvenile growth, and extreme cold are therefore priority nutritional stages.

PHLP schedules nutritional intervention from anticipated risk rather than visible weight loss. A practical decision combines seasonal forage forecast, recent body condition trajectory, physiological stage, and expected weather severity; the numerical trigger must be calibrated locally. Adjustment may include moving late-gestation or lactating females to a higher-priority group, increasing the proportion of oat hay or silage, adding a concentrate or urea–molasses block, or using a total mixed ration during a severe deficit [26,27]. Supplement amount should be stepped up gradually and reviewed against body condition, intake, fecal consistency, rumination, and feed cost. Forage reserves and emergency feeding plans should be established before winter or severe weather, and abrupt diet changes should be avoided to protect rumen stability.

### 6.3. Environmental Management and Animal Welfare to Reduce Stress

Cold, snow, large diurnal temperature shifts, intense ultraviolet radiation and long-distance grazing affect intake, rumination, reproduction and growth. These exposures may also alter health through neuroendocrine and immune pathways. Welfare research indicates that appropriate environments and opportunities for normal behavior can reduce stress and disease risk while supporting productivity and product quality [4,5].

Environmental and welfare conditions should be integral to health management. Wind protection, dry bedding, drainage, ventilation, daylight, and protected areas for neonates and late-gestation females should be matched to local climate. Transport decisions should be based on forecast temperature, wind chill, vehicle protection, journey duration, feeding status, and animal vulnerability rather than season alone. Experimental evidence from northern China indicates that low-temperature transport risk is greater in open vehicles, during longer journeys, and without appropriate pre-transport feeding [33]. Grazing schedules should likewise follow weather and rangeland condition, avoiding overgrazing, prolonged feed deprivation, and compulsory grazing during extreme weather.

### 6.4. Biosecurity, Disease Surveillance and Antimicrobial Stewardship

Biosecurity underpins animal health, public health and the long-term stability of livestock production. Alpine herds are dispersed, animals move frequently, and contact with wildlife may be common. Clinical treatment and routine vaccination alone cannot address this risk profile. A more complete system combines risk identification, barriers to transmission, continuous surveillance and standardized response [9].

Under PHLP, disease control expands from post-onset management to early risk identification. Examples include using local outbreak notifications before animal movement; checking introduced animals during a defined quarantine period; combining daily observation with deviations in intake, temperature, activity, or rumination; reviewing periparturient body condition trajectories; and intensifying parasite surveillance when season, grazing history, or fecal indicators suggest increasing risk [30,31]. These signals trigger assessment rather than automatic treatment. Core measures remain vaccination, quarantine, isolation, disinfection, complete records, biosecure carcass disposal, and veterinary confirmation before targeted therapy.

Reducing unnecessary antimicrobial use is another central objective of the PHLP framework. Inappropriate treatment selects for resistance and can compromise food and environmental safety [10,11]. Better environments, balanced nutrition and effective vaccination can reduce disease pressure and the need for treatment. Vaccines, probiotics, plant-derived additives or traditional Chinese veterinary medicines may offer additional options where evidence and local conditions support their use. Antimicrobial stewardship does not mean withholding treatment from animals that require clinical care. It requires veterinary diagnosis and, where appropriate, susceptibility testing to guide targeted therapy.

### 6.5. Nutrient Recycling and Low-Carbon Production for Ecological Co-Benefits

Livestock production must remain within the carrying capacity of alpine grasslands and surrounding ecosystems. Meat, milk, fiber, hides and manure are accompanied by methane and nitrous oxide emissions and by potential waste pollution [12,13]. Health-oriented production should therefore be assessed beyond the individual animal, including interactions with grassland, soil, water and climate.

PHLP emphasizes nutrient cycling and the environmental burden per unit of product. Housed and semi-housed units can integrate manure collection, composting, organic fertilizer use, clean water and wastewater separation, and biosecure treatment. Grazing systems require a different toolbox: stocking rates matched to assessed forage supply, rotational or deferred grazing, time-limited exclusion for restoration, reseeding where appropriate, and monitoring of plant cover and biomass. Controlled studies on the Qinghai–Tibet Plateau show that rotational grazing and context-specific grazing exclusion can improve biomass stability or degraded grassland recovery, although the optimal regime depends on grassland condition and duration [28,29]. These findings support adaptive grazing decisions rather than a universal prescription.

## 7. Potential Value, Current Limitations and Research Priorities

At present, the main value of PHLP is organizational and hypothesis-generating rather than technological. It connects established husbandry, veterinary, welfare, and ecological measures through an explicit sequence of forecast, detection, trigger, matched action, outcome assessment, and recalibration. Its contribution should therefore be judged by whether this decision architecture produces better predictions and decisions than reactive or calendar-based management, not by whether its individual components are new.

Conceptually, PHLP specifies different roles for adjacent frameworks. Proactive health supplies the anticipatory timing principle; preventive veterinary medicine supplies disease-specific measures; welfare supplies sentinel variables and safeguards; precision livestock farming supplies optional monitoring tools; sustainable livestock production defines resource and emission objectives; and One Health defines cross-system consequences. PHLP contributes the context- and stage-specific rule that orders these inputs and connects them to measurable feedback. 

Three propositions are testable. P1 predicts that trigger-based management will shorten warning-to-action time and reduce seasonal deterioration relative to reactive or fixed calendar-based management. P2 predicts that coordinated nutrition–environment–biosecurity actions will produce greater stability in functional indicators than isolated interventions. P3 predicts that joint animal–product–ecosystem decisions will reduce losses, treatment incidence, and environmental burden without worsening welfare, product-quality variability, or viability. PHLP would not be supported if decision timeliness and outcomes do not improve against an explicit comparator, proposed mediators do not change in the predicted direction, or gains are repeatedly offset by unacceptable costs or cross-system harms.

Implementation should be tiered rather than technology-maximal. A minimum package uses a seasonal risk calendar, reproductive-stage grouping, body-condition scoring, weather and forage information, disease and treatment records, and structured observation. An enhanced package adds group weighing, portable measurements, grazing location records, or targeted laboratory testing. An advanced package adds individual sensors and automated alerts where power, connectivity, maintenance, data management, and user training are reliable. In all tiers, priorities are ranked by risk severity, stage susceptibility, usable lead time, forage availability, actionability, labor demand, and expected value.

PHLP remains an early-stage conceptual framework. Its initial boundary conditions are alpine grazing or semi-grazing ruminant production; a consequential risk with usable lead time; at least one observable sentinel variable; a feasible intervention within that lead time; and local calibration of triggers. It is not a substitute for emergency clinical care, outbreak control, or statutory veterinary measures, and may add little when risk is wholly unpredictable or when no feasible action exists. Validation should use longitudinal cohorts, controlled or quasi-controlled comparisons, comparative farms, and multisite studies across elevations, species, seasons, and management intensity. Trials should report implementation fidelity, false alerts, missing data, maintenance, labor, capital and operating costs, cost per loss avoided, and gross margin. Economic incentives, extension support, shared equipment, and low-cost monitoring may be essential for smallholder and dispersed pastoral systems.

## 8. Conclusions

PHLP is proposed as a testable conceptual framework and a step toward a theory of proactive health-oriented livestock production, rather than as a mature universal theory. Its defining feature is a closed decision sequence linking context- and stage-specific risk, feasible sentinel signals, locally calibrated triggers, matched intervention, proposed functional mediators, and multi-level outcomes. Preventive veterinary measures remain indispensable within this sequence, while welfare, product quality, ecosystem pressure, and farm viability provide additional signals and constraints. For Tibetan sheep, yak, and other alpine ruminants, PHLP generates explicit comparisons against reactive or calendar-based management. Its scientific value will depend on prospective testing, transparent reporting of failed predictions and trade-offs, economic evaluation, and local calibration of indicators and triggers.

## Figures and Tables

**Table 2:** Candidate indicators for operationalizing and evaluating PHLP.

PHLP Component	Candidate Sentinel or Outcome	Measurement and Decision Use	Evaluation Criterion
Adaptive breeding	Survival, disease incidence, cold-season maintenance, fertility, growth, product quality	Multi-trait genetic or phenotypic recording; review correlated responses	Balanced gain without unacceptable loss of adaptation, diversity, or welfare
Nutrition and forage	Body-condition trajectory, live weight, forage biomass or quality, intake or rumination	Stage-specific monitoring before forecast deficit; adjust supplement or forage allocation	Smaller seasonal deterioration versus comparator at acceptable feed cost
Environment and welfare	Wind chill, shelter use, resting, feeding, locomotion, injuries, mortality	Structured observation or sensors; trigger shelter, route, timing, or stocking adjustment	Lower stress and welfare burden without transferring risk
Biosecurity and surveillance	Local alerts, quarantine findings, temperature, behavior, morbidity, treatment incidence	Risk-based surveillance and veterinary confirmation; escalate targeted response	Earlier appropriate action, fewer recurrent events, and no treatment delay
Rumen–gut and metabolic function	Rumination, fecal consistency, fermentation, selected metabolic or immune markers	Targeted sampling in validation studies; not necessarily routine on farm	Intermediate change in predicted direction
Grassland and low-carbon outcomes	Stocking pressure: forage supply, plant cover, biomass, nutrient return, product-normalized emissions	Seasonal rangeland assessment and product-based accounting	Lower burden without loss of grassland integrity or viability
Decision and economic performance	Warning-to-action time, adherence, false alerts, labor, capital and operating cost, gross margin	Audit every intervention cycle and compare with baseline or control	Timelier decisions and acceptable net benefit under the stated resource tier

## Data Availability

No new data were created or analyzed in this review. Data sharing is not applicable to this article.
